# Machine learning vs. ADM1: Reliable biogas prediction with minimal data requirements in full-scale plants

**DOI:** 10.1016/j.ese.2026.100662

**Published:** 2026-01-24

**Authors:** Sofia Tisocco, Sören Weinrich, Henrik Bjarne Møller, Alastair James Ward, Liam Kilmartin, Xinmin Zhan, Paul Crosson

**Affiliations:** a Civil Engineering, School of Engineering, University of Galway, Galway, H91 TK33, Ireland; b Teagasc Animal and Bioscience Research Department, Animal and Grassland Research and Innovation Centre, Dunsany, C15 PW93, Ireland; c Faculty of Energy · Building Services · Environmental Engineering, Münster University of Applied Sciences, Stegerwaldstraße 39, 48565, Steinfurt, Germany; d Biochemical Conversion Department, Deutsches Biomasseforschungszentrum gemeinnützige GmbH, Torgauer Straße 116, Leipzig, 04347, Germany; e Department of Biological and Chemical Engineering, Aarhus University, Blichers Allé 20, Tjele 8830, Denmark; f Electrical and Electronic Engineering, School of Engineering, University of Galway, Galway, H91 TK33, Ireland; g Ryan Institute, University of Galway, Galway, H91 TK33, Ireland; h MaREI Research Centre for Energy, Climate and Marine, Ryan Institute, University of Galway, Galway, H91 TK33, Ireland

**Keywords:** ADM1, Anaerobic digestion, Biogas technology, Feature importance, Machine learning, Parameter estimation

## Abstract

Anaerobic digestion harnesses microbial processes to convert organic wastes into renewable biogas, offering a sustainable pathway for energy production. In agricultural settings, biogas plants often co-digest livestock manure with crop residues, yet seasonal variations in feedstock quality introduce fluctuations that challenge process stability and yield optimization. Mechanistic models such as the Anaerobic Digestion Model No. 1 (ADM1) provide detailed biochemical simulations but require extensive substrate characterization, limiting their practicality for full-scale operations. Here we show that a simplified ADM1, alongside machine learning approaches—random forest and long short-term memory (LSTM) networks—achieves comparable accuracy in predicting daily biogas and methane production from a full-scale plant over 2023–2024. All models yielded Nash-Sutcliffe efficiencies above 0.78, with random forest excelling when incorporating feedstock quantities and maize silage volatile solids. While LSTM proved effective even with minimal inputs, it incurred a training time 141 times greater than ADM1, highlighting critical trade-offs in computational efficiency. These findings advance hybrid modelling strategies for real-time monitoring, enabling operators to balance predictive precision with data requirements to enhance renewable energy integration and agricultural sustainability.

## Highlights

• We compared a simplified ADM1 with RF and LSTM models to simulate biogas production at a full-scale plant.
• All three models achieved reliable predictive performance with Nash-Sutcliffe efficiency values above 0.78.
• LSTM enabled high-accuracy forecasting using only daily feedstock mass, reducing the need for chemical analyses.
• Maize silage was identified as the most critical input feature influencing biogas and methane production across models.
• LSTM training time was 141 times higher than ADM1, revealing a significant computational-accuracy trade-off.

## Graphical abstract

**Figure:**
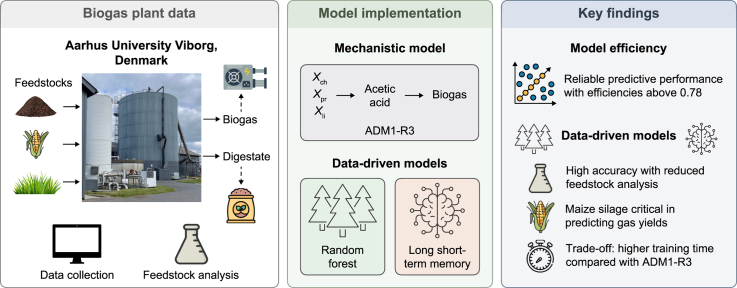

## 1 Introduction

Driven by ambitious greenhouse gas (GHG) emission reduction targets, anaerobic digestion (AD) of organic wastes and agricultural by-products is a widely acknowledged technology for sustainable waste management [1]. The AD process generates two primary outputs: biogas, a renewable energy source, and digestate, which can be utilized as an organic fertilizer, contributing to nutrient cycling within agricultural systems [2]. Livestock manure is a widely available feedstock for AD in agriculture. The AD process mitigates methane (CH_4_) and nitrous oxide (N_2_O) emissions from manure storage while enhancing the sustainability of waste management practices. However, due to low methane yields from mono-digestion of manure, co-digestion with other agricultural by-products is needed to enhance methane productivity and process stability [3]. In countries with diverse agricultural activities and high biogas production, such as Italy, Germany, and Denmark, the co-digestion of manure with other feedstocks, such as straw, grass, and crop residues, is widely adopted among biogas plants. This approach enhances the substrate's biochemical composition, improving methane yields and providing an efficient pathway for managing organic residues [4,5]. However, the seasonality of agricultural production, primarily driven by weather and management practices, leads to fluctuations in the quality and quantity of feedstocks available for AD throughout the year. These variations can affect the operational performance and biogas yield of AD plants. In this regard, implementing suitable models to support process monitoring and predict continuous biogas and methane production is crucial for optimizing AD performance. The Anaerobic Digestion Model No. 1 (ADM1) is a widely implemented model for simulating the different steps of the AD process [6] and has been applied to a wide variety of feedstocks and operating conditions [7]. However, given the extensive substrate characterization and numerous parameters required, its application in full-scale AD plants is limited. In this regard, Weinrich and Nelles [8] have developed four simplified versions of the ADM1 (ADM1-R1, ADM1-R2, ADM1-R3, and ADM1-R4), each of which incrementally reduces the number of reaction steps. This series of models aims to balance the need for precise AD outputs and the demands of real-time monitoring in large-scale AD processes. Tisocco et al. [9] implemented the ADM1-R3 to simulate full-scale co-digestion of cattle slurry and grass silage. The authors performed input variability analysis, including substrate composition (carbohydrate, protein, and lipid concentrations) and carbohydrate degradability, to assess their impact on biogas and methane production.

As an alternative to the ADM1, machine learning (ML) algorithms have been increasingly applied to simulate biogas and methane production using simplified AD input parameters, thereby reducing the need for extensive substrate characterization [10]. This approach enhances model applicability in scenarios where detailed feedstock composition data are unavailable while maintaining reliable predictive performance. For instance, Meola et al. [11] implemented a long short-term memory (LSTM) network combined with a genetic algorithm (GA) to optimize data preprocessing and hyperparameter estimation for simulating methane production in a full-scale reactor co-digesting rye whole crop silage and cattle manure. The model exhibited high efficiency with reduced input complexity, as the sensitivity analysis identified solid feed mass, feeding intervals, and volatile solids (VS) as the most influential parameters. Similarly, Yildirim and Ozkaya [12] applied five different ML algorithms to predict biogas production of a full-scale agricultural biogas plant using fundamental operational parameters, including total solids (TS), VS, temperature, pH, alkalinity, and volatile fatty acids (VFAs). Among the tested models, the random forest (RF) algorithm exhibited the highest predictive accuracy (*R*^2^ = 0.92). These studies highlight the potential of ML-based methodologies to achieve accurate predictions with simplified AD input parameters, thereby offering a viable alternative to mechanistic models such as ADM1.

The integration of ADM1 and ML models has been explored for predicting ADM1 kinetic parameters using various ML algorithms [13,14], and for comparing predicted outcomes [15]. For instance, Ge et al. [14] applied support vector machines (SVMs), artificial neural networks (ANNs), and RF algorithms to predict hydrolysis constants and biomass growth parameters using ADM1 to simulate food waste AD. Mathur et al. [15] utilized ML techniques to predict soluble chemical oxygen demand (sCOD) concentrations in effluent wastewater and compared these predictions with sCOD simulations generated by ADM1. However, direct comparisons of ADM1 and ML model performance in simulating continuous biogas and methane production from full-scale agricultural biogas plants remain underexplored. In particular, further research is needed to determine the minimum ML input requirements for achieving predictive accuracy comparable to ADM1. Additionally, evaluating the computational demand alongside predictive performance could provide a more comprehensive assessment of the model efficiency, thereby informing robust measurement protocols, improving data collection strategies, and enhancing predictive models for full-scale AD systems.

In this context, this study aimed to: (1) apply a simplified ADM1 as well as two ML algorithms, RF and LSTM, to simulate the biogas and methane production at a full-scale biogas plant co-digesting different agricultural feedstocks, comparing their predictive accuracy and robustness in representing process dynamics; (2) identify the feedstocks and AD parameters that most affect simulated biogas and methane production in the ML models; and (3) assess the computational demand of the selected models to provide a comprehensive understanding of their performance.

## 2 Materials and methods

### 2.1 Plant description and data sets

The models were applied to simulate a full-scale biogas plant co-digesting cattle manure, deep litter, maize silage, grass silage, and other agricultural feedstocks (Supplementary Fig. S1) at the Aarhus University (AU) biogas plant in Foulum, Denmark. The biogas plant consists of two continuously stirred-tank reactors: a heated primary digester under thermophilic conditions (53 °C) with a reactor volume of 1200 m^3^, and a heated secondary digester under thermophilic conditions (48 °C) with a reactor volume of 30 m^3^. The hydraulic retention time (HRT) of the primary reactor ranges from 13 to 15 days, while the secondary reactor has an HRT of 30 days, resulting in a total HRT of approximately 45 days for the entire AD process. Gas yields are measured in each rector. The biogas in the tanks’ headspace is transferred to a 1500 m^3^ gas storage bag before being utilized by a combined heat and power (CHP) engine to produce electricity and heat (maximum capacity of 538 and 672.5 kWh, respectively). The digestate produced is stored in a 2355 m^3^ tank and subsequently spread across different farmlands as an organic fertilizer. For the present study, data from the primary digester were used for simulation, as it produced most of the biogas and provided more detailed information. Biogas production is adjusted to meet the heat demand of AU Foulum, which varies seasonally. Consequently, the organic loading rate (OLR) is adjusted accordingly, reaching its lowest levels during the summer months from June to September (Supplementary Fig. S2) and with an average organic loading rate (OLR) of 6.1 kg VS m^−3^ d^−1^. Two years of data, 2023 and 2024, were used for modelling, split into calibration and validation sets. This chronological division was chosen to preserve the seasonal patterns and intra-annual fluctuations in biogas production. The feedstocks supplied to the AD plant during these years included livestock slurry, deep litter, maize and grass silage, as well as other agricultural feedstocks (Supplementary Fig. S1).

### 2.2 Measurement methods

#### 2.2.1 Input measurements

Daily available data included quantity of each feedstock fed (t d^−1^), biogas production (m^3^ d^−1^), and electricity and heat produced (kWh). Characteristic analyses from feedstocks included weekly to monthly measurements for TS, VS, ammonium nitrogen (NH_4_-N), total nitrogen (N), fibre content (XF), and VFAs (Table 1; Supplementary Table S1). TS of feedstocks was measured by heating samples to 105 °C for 24 h, and VS was further measured by heating the dried sample at 550 °C for 2 h [16]. NH_4_-N was measured photometrically in duplicate using Spectroquant kits and a NOVA 60 spectrophotometer [17]. Total N was measured through the Kjeldahl method [16]. VFAs (acetic, propionic, butyric, and valeric acid) were measured using an Agilent 6850 gas chromatograph (Agilent Technologies, CA, USA) equipped with a flame ionization detector and helium as the carrier gas. A DB-1 column was employed, with the injection port and detector temperatures set at 250 and 300 °C, respectively [17]. Crude proteins (XP) of feedstocks were indirectly calculated by multiplying the organic N (total N minus inorganic N) by 6.25 [16]. Crude lipids (XL) values were obtained from the literature in the absence of available measurements (Table 1).

**Table 1:** Feedstock characterization implemented in this study^a^.

Parameter (unit)	Cattle manure	Deep litter	Maize silage	Grass silage	Meadow grass	Fresh grass	Straw	Animal feed waste
TS (% FM)	3.6 ± 1.4	30.7 ± 1.2	30.1 ± 1.7	31.9 ± 0.7	81.8 ± 0.0	10.7 ± 0.0	85.0 ± 0.0	32.8 ± 3.1
VS (% TS)	75.1 ± 6.8	85.0 ± 0.7	96.7 ± 0.2	87.7 ± 0.4	91.9 ± 0.0	88.9 ± 0.0	94.2 ± 0.0	90.0 ± 0.1
XA (g per kg TS)	249.4	150.0	33.4	123.1	80.6	111.2	57.5	100.0
XC (g per kg TS)^b^	586.6	699.3	846.1	731.0	782.2	713.5	884.1	731.2
XP (g per kg TS)	124.0	123.7	76.0	116.9	108.2	146.3	42.5	117.8
XL (g per kg TS)	48.5^c^	27.0^d^	44.5^c^	29.0^e^	29.0^e^	29.0^e^	15.9^f^	51.0^g^
NH_4_-N (g L^−1^)	1.08	1.20	0.76^c^	7.28	11.7	2.50	0.31^h^	1.10
Acetic acid (g L^−1^)	2.57	1.75	9.98^c^	17.4^i^	18.4^i^	16.3^i^	0.13^h^	9.98^c^
Butyric acid (g L^−1^)	n.d.	0.14	0.09^c^	n.d.	2.4^i^	n.d.	n.d.	0.09^c^
Propionic acid (g L^−1^)	0.99	0.27	0.28^c^	n.d.	n.d.	n.d.	n.d.	0.28^c^
Lactic acid (g L^−1^)	n.d.	n.d.	n.d.	46.4^i^	n.d.	45.9^i^	n.d.	n.d.
Valeric acid (g L^−1^)	0.11	n.d.	0.01^c^	n.d.	n.d.	n.d.	n.d.	0.01^c^
XF (g per kg TS)	n.d.	n.d.	211.4	244.4	n.d.	267.4	n.d.	200.0
ADF (g per kg TS)	n.d.	471.0	231.8	417.0	n.d.	n.d.	n.d.	n.d.
NDF (g per kg TS)	n.d.	641.4	423.1	662.7	n.d.	n.d.	n.d.	n.d.
ADL (g per kg TS)	n.d.	123.2	26.5	74.1	61.8^j^	n.d.	136.9^k^	n.d.
DQ_XC_ (% XC)	69^c^	n.d.	n.d.	n.d.	n.d.	n.d.	n.d.	n.d.

Notes.

a Abbreviations: TS, total solids; FM, fresh matter; VS, volatile solids; XA, crude ash; XC, crude carbohydrates; XP, crude proteins; XL, crude lipids; NH_4_−N, ammonium nitrogen; XF, crude fiber; ADF, acid detergent fiber; NDF, neutral detergent fiber; ADL, acid detergent lignin; DQ_XC_, degradability quotient of crude carbohydrates; n.d., not determined. Values are from direct measurements unless otherwise indicated. Where available, data are shown as mean ± standard deviation (s.d.); entries without an accompanying s.d. are reported as single values from the underlying source(s).

b XC was calculated as XC = 1000 − XA − XP − XL (all in g per kg TS) [18].

c Weinrich et al. [18].

d Huber et al. [19].

e Tisocco et al. [9].

f Average from Kaparaju et al. [20]; Sträuber et al. [21].

g Mogensen and Kristensen [22].

h Kaparaju et al. [20].

i Reported in the source as g per kg TS (not g L^−1^) and reproduced here as reported.

j Feng et al. [23].

k Average from Lin et al. [24]; Rahman et al. [25].

#### 2.2.2 Outputs measurements

The measured outputs included daily biogas production rate (m^3^ d^−1^) and weekly CH_4_ content in the biogas from the primary reactor, along with monthly data on NH_4_-N concentrations and pH in digestate samples taken from the primary reactor. The pH was measured with a Knick 911 pH meter [17]. Measurement methods for NH_4_-N are described in Section 2.2.1.

### 2.3 Models implementation

#### 2.3.1 ADM1-R3

The ADM1-R3 was applied [8] to simulate biogas and methane production of a full-scale agricultural biogas plant. The model was implemented in MATLAB (The MathWorks, Inc., USA) and is available on GitHub (soerenweinrich/ADM1).

***Model inputs.*** Crude carbohydrates (XC), proteins (XP), and lipids (XL) were transformed into ADM1 inputs (*X*_ch_, *X*_pr_, and *X*_li_) considering the TS and their degradability [9]. For XP and XL, 100 % degradability was assumed [26], whereas the *X*_ch_ for each feedstock was calculated using its degradability quotient (DQ_XC_), acid detergent lignin (ADL), or crude fibre (XF) content, depending on the available measurement (Supplementary Table S2).

***Parameter estimation.*** During the AD of lignocellulosic biomass, hydrolysis is the rate-limiting step [27]. During this study, hydrolysis rates for carbohydrates (*k*_ch_), proteins (*k*_pr_), and lipids (*k*_li_) were estimated by minimizing the root-mean-square error (*RMSE*) between simulated and measured biogas production [9,18]. The optimization was performed using the MATLAB function *fmincon* (The MathWorks, Inc., USA), with lower and upper bounds set at 0.001 and 10, respectively, to ensure reasonable parameter estimates [18]. The remaining model parameters were set to default values [9]. The parameter estimation process utilized data from 2023, and the resulting parameters were subsequently applied to the 2024 data set for validation.

#### 2.3.2 Machine learning algorithms

We selected RF and LSTM as the ML algorithms for this study, as they are considered robust approaches for simulating full-scale AD applications [28] and represent fundamentally different modelling concepts and computational structures [29]. The algorithms were applied to simulate biogas and methane production using various feedstocks and AD parameter combinations as model inputs. We used data from 2023 for model training, and data from 2024 for model validation (Section 2.1). The algorithms were implemented in Python 3.13 using different libraries, including *NumPy* [30] and *Pandas* [31] for data manipulation and preprocessing, *Scikit-Learn* (Pedregosa et al., 2011) for RF model implementation and hyperparameter optimization, and *TensorFlow* [32] for LSTM development and training.

***Random forest.*** RF is an ensemble learning method that constructs multiple independent decision trees during training and combines their predictions to enhance accuracy and robustness [33]. The simulation results represent the average predictions of individual decision trees, thereby enhancing the model's robustness in capturing complex, non-linear patterns in the data [34].

***Long short-term memory network.*** LSTM is a type of recurrent neural network (RNN) designed to learn long-term dependencies in sequential data. LSTMs are equipped with memory cells and gating mechanisms (input, forget, and output gates), which enable them to retain and selectively update relevant information over time, making them particularly suitable for simulating time-series data [35].

***Hyperparameter estimation.*** To enhance prediction accuracy, hyperparameter optimization for both LSTM and RF models was performed using *GridSearchCV* from the *Scikit-Learn* package [36]. *GridSearchCV* searches over a previously specified hyperparameter grid using cross-validation to identify the best-performing model configuration based on a scoring metric, with the optimal hyperparameters determined by minimizing the mean squared error (MSE) [11]. The RF hyperparameter grid included estimators, tree depth, split and leaf sample thresholds, and maximum features, while LSTM tuning covered layer units, dropout rate, optimizer, batch size, and epochs (Supplementary Table S3).

**Fig. 1:**
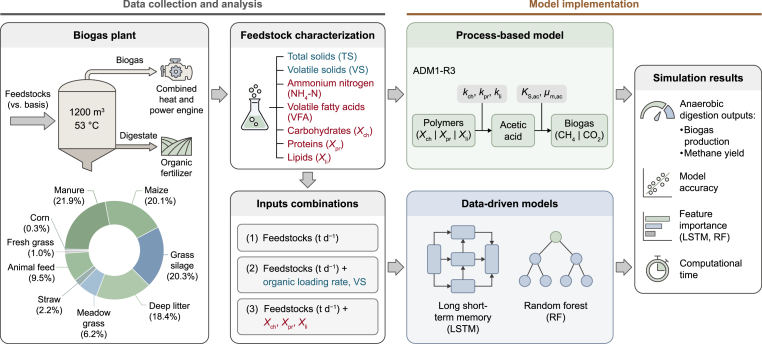
Overview of the study workflow, from biogas-plant data acquisition and feedstock characterization to model implementation and evaluation. Feedstock/input variables are colour-coded by measurement complexity: blue, routine chemical analyses (e.g., total solids and volatile solids); red, advanced measurements (e.g., NH_4_-N, volatile fatty acids, and biochemical fractions). Three input-feature combinations were constructed and used to run a process-based anaerobic digestion model (ADM1-R3) and train data-driven models (long short-term memory and random forest), which were evaluated against anaerobic digestion outputs (biogas production and methane yield), together with model accuracy, feature importance, and computational time.

***Features for ML algorithms.*** Different input combinations were incorporated into the ML algorithms to evaluate the importance of AD parameters in predicting biogas and methane production from a full-scale agricultural biogas plant. The features included individual daily feedstock quantities (t d^−1^), OLR (kg VS m^−3^ d^−1^), ADM1 inputs (sum of *X*_ch_, *X*_pr_, and *X*_li_, in kg d^−1^), and the VS flow (t VS d^−1^) of maize silage, given its substantial VS proportion in the feedstock inputs (Fig. 1; Supplementary Fig. S1). This approach aimed to identify the most influential feedstock-related factors for accurate simulation of biogas and methane production and to assess the relevance of extensive substrate characterization (i.e., carbohydrates, proteins, and lipids) for monitoring full-scale agricultural biogas plants. While substrate characterization is essential for ADM1, its relevance for ML models is less clear, as they rely on data patterns rather than explicit biochemical pathways [37]. The Pearson correlation coefficient was calculated to assess linear relationships between input variables and biogas production using the *Seaborn* library in Python [38]. Additionally, feature importance was assessed to quantify each input variable's contribution to the model output. Shapley additive explanations (SHAP) values were implemented to interpret the influence of individual input features on the model's predictions. SHAP analysis is based on cooperative game theory and calculates the marginal contributions of each feature to the overall model's outputs across all possible feature combinations [39,40]. This approach provides a comprehensive assessment of each feature's impact on the model, offering both global and local interpretations of implemented ML models.

### 2.4 Model efficiency

The Nash–Sutcliffe efficiency coefficient (*NSE*; [41]) was implemented to assess the statistical differences between measurements and simulation results (equation (1)):

(1) $$NSE=1-\frac{\sum_{i=1}^{n}{\left(x_{i}-y_{i}\right)}^{2}}{\sum_{i=1}^{n}{\left(x_{i}-{\bar{x}}_{i}\right)}^{2}}$$

where $x_{i}$ represents the measured values and $y_{i}$ denotes the simulation results. A negative *NSE* indicates that model predictions are less accurate than the mean of observed values, while a value of 0 reflects equivalent performance to the mean. Positive values (up to 1) reflect increasing predictive ability relative to the mean. To evaluate both the detail in simulations and broader system representation [9], daily and weekly *NSE* values were calculated. Daily *NSE* compared each simulated daily value with its corresponding observed value, while weekly *NSE* compared the weekly production sum of simulated and observed values.

### 2.5 Computational demand

Simulation models can present challenges due to the trade-off between accuracy and the computational effort required for model execution [42]. To assess the computational demand of the implemented models, training and testing simulation times for each model were recorded separately. For ADM1-R3, execution time was measured using the *tic-toc* function in MATLAB for both calibration (2023) and validation (2024) data. For RF and LSTM, the performance counter was imported from the time module in Python to track the start and end times of both training and testing phases. Additionally, random-access memory (RAM) usage was monitored for training and testing phases of the models to provide a more comprehensive assessment of their computational efficiency [43,44]. To evaluate RAM consumption of the ML models and ADM1-R3, the resident set size was recorded at multiple intervals during both the training (2023) and validation (2024) simulations. For the ML models, memory usage was measured at various points throughout the training and testing phases, allowing calculation of maximum (‘peak’) and average memory consumption using the *Psutil* library in Python.

## 3 Results and discussion

A full-scale biogas plant co-digesting agricultural feedstocks under thermophilic conditions was modelled, implementing a simplified ADM1 and two ML algorithms (RF and LSTM). The data from the biogas plant covered two years (2023 and 2024) of continuous operation, during which no process failures or inhibitions were observed.

### 3.1 Comparison of model performance and efficiency

**Fig. 2:**
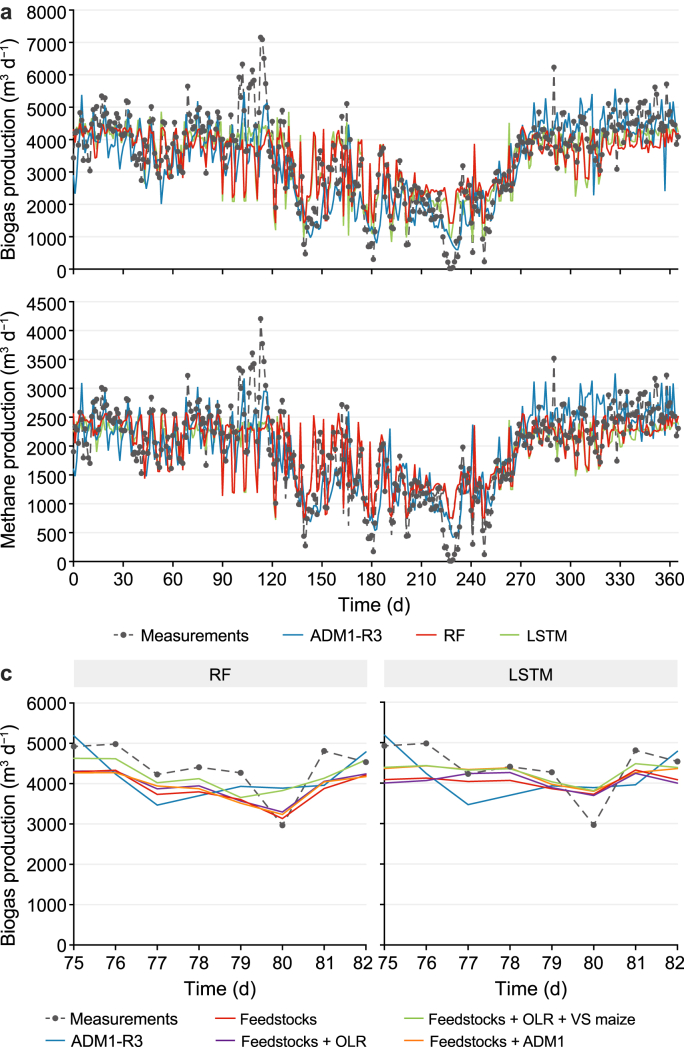
Model validation and sensitivity to input-feature sets. **a**, Validation of biogas and methane production in 2024 using ADM1-R3, random forest (RF), and long short-term memory (LSTM). Inputs: feedstocks, organic loading rate (OLR), and volatile solids (VS) from maize silage. **b**, Impact of different input combinations on RF and LSTM simulations.

**Fig. 3:**
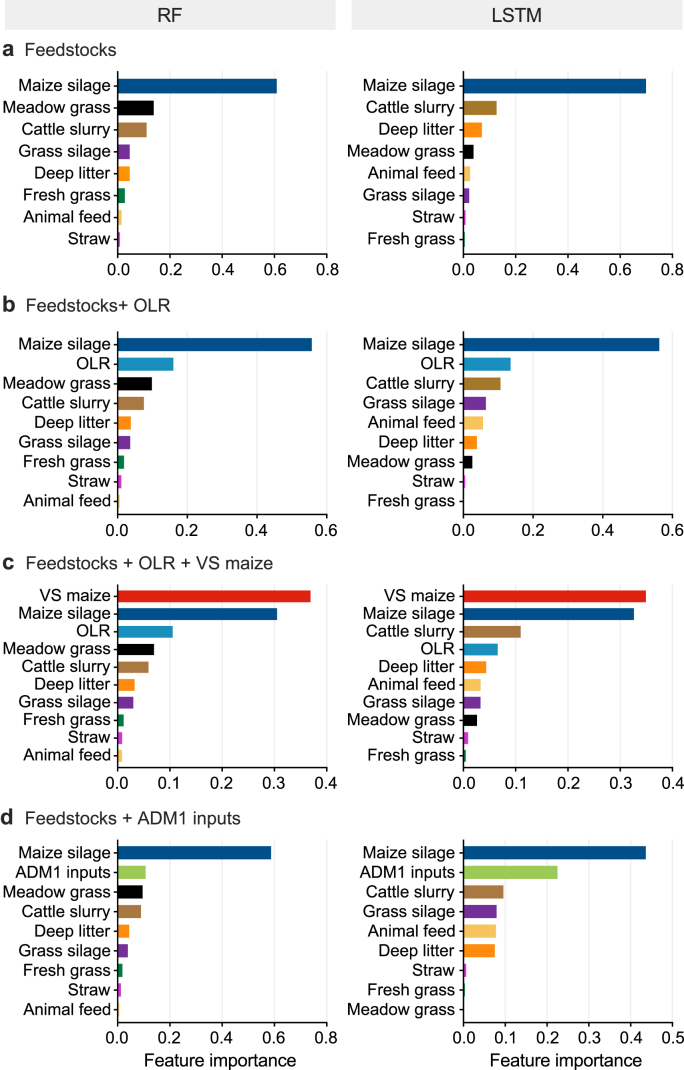
Shapley additive explanations (SHAP)-based feature importance for biogas production. Normalized mean absolute SHAP values for the random forest (RF; left) and long short-term memory (LSTM; right) models, computed across the 2024 validation period. Input-feature sets include: **a**, Feedstock quantities (t d^−1^); **b**, Feedstocks plus organic loading rate (OLR; kg VS m^−3^ d^−1^); **c**, Feedstocks plus OLR and maize volatile solids (VS maize; t VS d^−1^); **d**, Feedstocks plus aggregated ADM1 composition inputs (kg d^−1^).

Simulation results from biogas and methane production from the three models indicated adequate performance in depicting measurements (Fig. 2), with comparable NSE values (Table 2). Simulation curves from RF and LSTM exhibited higher similarity, even though LSTM demonstrated an improved ability to capture peaks (i.e., days 165 and 197) and drops (i.e., days 139 and 241) compared to RF. The similarity between ML simulations and ADM1-R3 was likely due to their greater dependence on feedstock patterns over the year [45] rather than AD kinetics. Considering that biogas production throughout the year at the biogas plant was adjusted based on heat demand, summer months (days 152–274) had the lowest feedstock quantity fed (thus lower OLR) and biogas production (Fig. 2; Supplementary Fig. S2). ADM1-R3 best captured both the drops and peaks in biogas and methane production, likely due to its explicit modelling of organic content and detailed representation of nutrient availability in substrate degradation. Among all input features in the ML models, maize silage had the greatest impact on simulation outcomes, as indicated by the feature importance analysis (Fig. 3 and Section 3.3) and maize feeding intervals. For instance, when maize silage was not fed in certain days from days 90–120, both ML models exhibited an abrupt drop in biogas and methane production, whereas ADM1-R3 maintained more stable gas yields (Fig. 2a). These daily fluctuations in feedstock amount also suggest that for full-scale biogas plants, weekly biogas production monitoring may be a more suitable frequency interval, considering daily variations may not be as accurate and representative of the overall AD system. This was further reflected in the higher weekly NSE values observed across all models (Table 2).

**Table 2:** Predictive skill on the 2024 test set. Daily and weekly Nash–Sutcliffe efficiency (*NSE*) for biogas and methane production across process-based and data-driven models, evaluated against measurements.

Model family	Model and input combinations	Temporal aggregation^a^	*NSE* (biogas)	*NSE* (methane)
Process model	ADM1-R3	Daily	0.55	0.56
		Weekly	0.82	0.84
Random forest	Feedstocks	Daily	0.56	0.54
		Weekly	0.72	0.70
	Feedstocks + OLR	Daily	0.58	0.57
		Weekly	0.74	0.72
	Feedstocks + OLR + VS maize	Daily	0.60	0.59
		Weekly	0.82	0.79
	Feedstocks + ADM1 inputs	Daily	0.59	0.56
		Weekly	0.73	0.70
Long short-term memory	Feedstocks	Daily	0.62	0.59
		Weekly	0.81	0.77
	Feedstocks + OLR	Daily	0.63	0.61
		Weekly	0.82	0.78
	Feedstocks + OLR + VS maize	Daily	0.62	0.61
		Weekly	0.81	0.78
	Feedstocks + ADM1 inputs	Daily	0.61	0.63
		Weekly	0.78	0.80

Note:*NSE* was computed by comparing measured and simulated production time series. “Daily” denotes day-by-day *NSE* using daily values, whereas “Weekly” denotes *NSE* computed on weekly totals (weekly sums).

### 3.2 Training data and parameters estimation

The estimated hydrolysis rates for carbohydrates, proteins, and lipids from ADM1-R3 (Table 3) lied close to the default values reported for the AD of lignocellulosic materials [9,18], which supports the validity of the parameter estimation process and results obtained. For ADM1-R3, the *NSE* was higher for the validation data set (2024) than for the calibration data set (2023) (Supplementary Table S4; Supplementary Fig. S3), likely due to greater use of well-characterized feedstocks such as cattle slurry, maize silage, grass silage, and deep litter (Table 1; Supplementary Table S1). In contrast, 2023 involved a higher proportion of less commonly used feedstocks, such as straw and meadow grass, for which limited feedstock quality data were available. Consequently, the characterization of these feedstocks, particularly the degradability of carbohydrates [9], may have been overestimated, leading to higher simulated biogas outputs than measurements (Supplementary Fig. S3).

**Table 3:** Estimated ADM1 hydrolysis parameters and optimized machine-learning hyperparameters (2023 calibration). Parameter and hyperparameter values obtained from optimization using 2023 data. For the random forest and LSTM models, results correspond to the Feedstocks + OLR + VS maize input set.

Model	Parameter/hyperparameter	Optimized value
ADM1-R3	Hydrolysis rates of carbohydrates (*k*_ch_)	0.25
	Hydrolysis rates of proteins (*k*_pr_)	0.23
	Hydrolysis rates of lipids (*k*_li_)	0.12
Random forest	Number of estimators	500
	Maximum depth of trees	10
	Minimum samples to split	15
	Minimum samples per leaf	2
	Maximum features for splits	Square root (sqrt)
Long short-term memory	Number of units	128
	Dropout rate	0.1
	Optimization algorithm	Adaptive moment estimation (Adam)
	Batch size	16
	Epochs	50

Hyperparameters from RF and LSTM were estimated through *GridSearchCV*. For RF, an ensemble of 500 estimators with a maximum depth of 10 was used, requiring at least 15 samples for node splitting and at least 2 samples per leaf (Table 3). Similarly, Wang et al. [46] implemented an RF algorithm with 300 estimators and a depth of 10 to predict methane yields from anaerobic digestion of straw, incorporating various AD parameters, including temperature, OLR, and microbial community composition. Their model achieved comparable predictive performance (*R*^2^ = 0.69) to the efficiencies observed in this study. For LSTM, 128 units with a dropout rate of 0.1 were implemented. The Adam optimization algorithm was selected to enhance convergence efficiency [11], with a batch size of 16 and 50 training epochs. The LSTM hyperparameters were kept within narrower ranges than in other studies that used complex optimization algorithms, such as GA and particle swarm optimization (PSO) [11,46]. This simplified approach guaranteed an effective balance between hyperparameter optimization and computational efficiency [43].

In contrast to ADM1-R3, the *NSE* obtained by the ML algorithms during training (2023) was higher than during validation (2024) (Supplementary Tables S2 and S4), indicating effective pattern recognition in the training set but reduced generalization to unseen data. Notably, a larger gap was observed between training and testing performance for RF, whereas LSTM exhibited relatively stable efficiency across both data sets. This observation was further corroborated by the *RMSE* calculated for each model and data set (Supplementary Table S5), with RF exhibiting the largest gap between training and testing performance. Differences in the efficiency of training and testing models within RF have been reported previously [45,47]. This discrepancy may be attributed to the fundamental differences in how these models learn patterns from data. RF relies on decision trees, which can become highly specialized to training data, potentially leading to overfitting [48]. Conversely, LSTM networks, designed for time-series data, use gated architectures to capture and update information over long sequences [29,35,37], enabling more accurate modelling of temporal biogas and methane fluctuations driven by seasonal feedstock changes. Additionally, for a more direct comparison with the ADM1-R3, only one year of data was used for ML training (representing 50 % of the total data set), whereas a common range for training data sets is around 75 % [11,12,47]. Therefore, using larger training data sets should be considered to enhance model performance and improve generalization to unseen data [46,49].

### 3.3 Model inputs and impact on simulation results

Different input combinations were evaluated within the ML models, and feature importance was further calculated to assess the impact of AD parameters on simulation results. The ML algorithms achieved comparable performance to ADM1-R3 using only individual feedstock quantities (t d^−1^) as input features (Fig. 2b), although the LSTM achieved a higher *NSE* than RF (Table 2). SHAP values were calculated to assess the contribution of each input feature to the model outputs. To facilitate model comparison and assess the relative contribution of each feature, normalized mean absolute SHAP (|SHAP|) values were computed by dividing each feature's mean |SHAP| value by the total sum across features [50]. The mean |SHAP| value represents the average magnitude of a feature's impact on the model output, irrespective of direction, offering insights into their feature importance [51]. SHAP analysis identified maize silage as the most influential feature for predicting biogas (Fig. 3) and methane production (Supplementary Fig. S4) in both algorithms, likely because it contributed most to the total organic content in 2024 (Supplementary Fig. S1b). This outcome aligns with ADM1's biochemical framework and the fact that a higher organic content of input feedstocks drives increased biogas production, highlighting consistency between data-driven feature importance and mechanistic process understanding.

De Clercq et al. [49] also observed high model efficiency when using only feedstock quantities as inputs for RF and extreme gradient boosting (XGBoost) to simulate biomethane production from an industrial-scale AD plant co-digesting food waste and agricultural feedstocks. In their study, RF achieved an *R*^2^ ranging from 0.88 to 0.82 when predicting biomethane production 1–40 days ahead based on feedstock inputs. In our study, RF achieved an *NSE* value of 0.70 for biomethane production when utilizing only feedstock quantities as input features (Table 2), over a continuous period of 365 days (Fig. 2). The authors emphasized the trade-off between obtaining additional substrate characterization to potentially enhance model efficiency and the associated economic and logistical costs. They further argued that the lower frequency of these measurements compared to the more regular monitoring of fresh inputs may not provide optimal conditions for accurate model performance. Thus, feature selection should be carefully considered, taking into account the total number of features and the sample size per feature to mitigate overfitting [52]. Input features are often assessed using advanced statistical methods, such as principal component analysis (PCA) [12,45], to identify the most influential parameters affecting output variability. Additionally, Pearson correlation matrices are computed to assess correlations between input features and AD outputs [37,45].

**Fig. 4:**
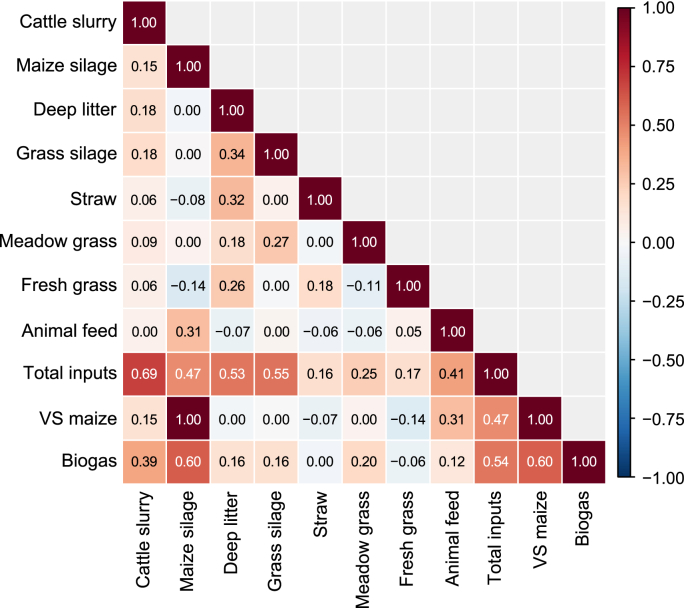
Pearson correlation matrix structure between input features and biogas production. Heat map shows pairwise Pearson correlation coefficients (*r*; numbers) among daily feedstock inputs, derived aggregate variables, and biogas production; colours indicate *r* from −1 to 1. Feedstock inputs are expressed as wet mass (t d^−1^); Total inputs is the sum across all feedstocks; VS maize denotes the maize volatile-solids input (kg VS d^−1^). Biogas denotes daily biogas production (m^3^ d^−1^). Correlation coefficients are rounded to two decimal places, and coefficients with |*r*| < 0.05 are omitted and displayed as 0.

During this study, feedstock was the main AD parameter that showed the greatest variation, whereas other AD operational parameters (e.g., temperature) remained constant or were not measured (e.g., microbial composition). Consequently, different input combinations related to feedstock characterization were evaluated. These combinations included basic feedstock characterization parameters, such as VS quantified through the OLR, as well as more detailed ADM1-derived parameters, including *X*_ch_, *X*_pr_, and *X*_li_. A previous study by the authors [9] showed that carbohydrate, protein, and lipid variations had a limited impact on biogas and methane simulations using ADM1 [9]. Nevertheless, the current study included both basic and detailed substrate characterization into the ML algorithms to assess their influence on biogas and methane simulations. VS flow (t VS d^−1^) from maize silage was also included in the input features alongside feedstock quantities and OLR, given the significant impact of maize silage on biogas production (Fig. 3, Fig. 4). Among the evaluated feedstock characteristics, TS and VS from cattle slurry exhibited the greatest variability (Table 1), likely due to the large volume of slurry fed into the AD plant (Supplementary Fig. S1a). These fluctuations partially contributed to the variation observed in OLR and ADM1-derived inputs, including *X*_ch_, *X*_pr_, and *X*_li_. Nevertheless, stronger correlations were observed between these parameters and feedstocks of higher quality, such as grass and maize silage, and deep litter (Fig. 4). This was likely attributable to their higher organic content (TS, VS, carbohydrates, proteins, and lipids) compared to cattle slurry, despite slurry being supplied in larger fresh proportions and thus showing a strong correlation with total feedstock quantity (Fig. 4). These findings also suggest that characterizing feedstocks solely by their individual concentrations (i.e., kg VS m^−3^) would not be adequate for this study, as changes in these parameters were less representative of biogas and methane production patterns compared to total flow variations. This was due to either low correlations with gas yields (i.e., cattle slurry) or minimal variation within these parameters (i.e., TS and VS from maize silage, grass silage, and deep litter; Table 1). Therefore, total VS flow (t VS d^−1^), as well as the sum of carbohydrates, proteins, and lipids (kg d^−1^), were selected as input features.

Incorporating OLR and VS flow from maize silage into the ML models achieved the highest NSE for methane production in both algorithms and for biogas production in RF (Fig. 2; Table 2). In contrast, biogas simulations from LSTM remained relatively unchanged with the incorporation of new input features. Notably, Fig. 4 demonstrated a correlation of 1 between fresh matter (t d^−1^) and VS flow (t VS d^−1^) from maize silage, attributable to the consistent TS and VS content of this feedstock throughout the year. The main difference between fresh matter and VS flow lay in the water content, considering that most TS content was VS (Table 1). The water content is positively correlated with biogas production, as it facilitates the AD process, particularly during the initial hydrolysis and acidogenesis stages [53]. Therefore, the information gained by indirectly incorporating water content (through the difference between fresh and VS flows) may help explain the improved results achieved by including VS flow from maize silage as an input feature, despite potential multicollinearity among input variables. Finally, because *X*_ch_, *X*_pr_, and *X*_li_ variability also depended on feedstock amount, their fluctuations followed the same pattern as OLR. Thus, no significant difference was observed between OLR and ADM1-derived input combinations for the ML algorithms (Fig. 2, Table 2). This suggests that, for the present data sets, no additional gain was achieved by incorporating more detailed feedstock chemical analyses into the ML models.

Li et al. [47] assessed the impact of food waste characterization on ML simulations using different algorithms such as RF, XGBoost, and ANN. They found that combining feedstock quantities (t d^−1^) with feedstock chemical composition and digester properties (i.e., TS in g kg^−1^, VFAs, and chemical oxygen demand in mg L^−1^) improved RF model efficiency by up to 54 % compared to utilizing feedstock quantities alone.

Similarly, Salamattalab et al. [54] implemented an LSTM algorithm combined with GA to simulate biogas production from a full-scale AD process treating wastewater and assessed different ML input combinations. However, they found that combining wastewater characteristics (i.e., COD, total suspended solids, and total N in mg L^−1^) with digester flow rates (m^3^ d^−1^) did not substantially improve the model efficiency compared to utilizing flow rates alone. In the present study, incorporating feedstock characterization based on flow rates (i.e., kg VS d^−1^) rather than concentrations (i.e., kg VS m^−3^) enhanced RF model predictions of biogas and methane yields by 14 % and 13 %, respectively (Table 2). However, in alignment with Salamattalab et al. [54], this incorporation had minimal impacts on LSTM simulations (Table 2), suggesting that, at least in terms of input features, the model had already achieved high efficiency by incorporating only feedstock amount (t d^−1^). Nevertheless, further improvements in model structure, such as optimizing hyperparameters or data preparation procedures, could further enhance overall model efficiency [11,28]. Differences in model performance between RF and LSTM when incorporating additional features can be attributed to their distinct learning mechanisms. RF constitutes a tree-based ensemble method that improves prediction accuracy by selecting the most informative features at each split, thus refining decision boundaries. In contrast, LSTM networks are designed to capture sequential dependencies over time, meaning that additional input features may not enhance their predictive capability unless they introduce new temporal patterns [29,37]. This limitation was evident for OLR and ADM1-derived inputs, as their values were largely dependent on feedstock amounts and did not introduce new temporal dynamics. Similarly, Meola and Weinrich [28] noted that, for data sets with no observed process inhibition, time-dependent variables, such as the hour of feeding, were more influential for LSTM predictions than feedstock characteristics beyond VS content.

In summary, ML-based simulations of biogas and methane production from the co-digestion of agricultural feedstocks have produced variable results depending on the AD system modelled. In systems with a diverse range of agricultural waste feedstocks, such as in the present study and De Clercq et al. [49], accurate model performance can be achieved by relying only on individual feedstock quantities. This approach proved effective because the biogas plant received a diverse range of feedstocks, which naturally provided sufficient variability for model training. Although incorporating additional quality parameters (VS, *X*_ch_, *X*_pr_, and *X*_li_) generally enhanced *NSE*, these input features were also correlated with feedstock quantities (Fig. 4). Conversely, studies involving more homogeneous feedstock compositions, with only one or a few feedstock types [47,54,55], reported improved model accuracy when feedstock characteristics were expressed as concentrations (i.e., mg COD L^−1^). In these scenarios, the limited heterogeneity of feedstock types required more detailed feedstock characterization to accurately represent system dynamics. Therefore, the choice of input features is likely to depend on the operating conditions and feedstock diversity of the AD plant. Furthermore, due to the ‘black-box’ nature of ML algorithms, the impact of feedstock quality on biogas production may not be as clear as in ADM1, where AD biochemical pathways are explicitly modelled. Since ML models are heavily dependent on historical data, changes in feedstock composition or feeding mode can significantly affect model performance. Consequently, mechanistic models or biochemically-based methods remain essential for evaluating the effects of new feedstocks and operating conditions on AD performance. It should be noted that other operational parameters, such as NH_4_-N, VFAs, and alkalinity, are critical for comprehensive monitoring of the AD process, and their inclusion in the ML models could potentially enhance predictive performance. Thus, their exclusion represents a limitation of the present study, which focused on assessing the influence of feedstock characterization on biogas and methane production.

### 3.4 Computational demand

To evaluate the computational complexity of the implemented models, both time and memory consumption during training and testing were recorded. Among the evaluated models, LSTM exhibited the longest training runtime, averaging 552.8 s, 11 times that of RF (45.9 s) and 141 times that of ADM1-R3 (3.9 s) (Table 4). The standard deviation of LSTM's training time also reflects high variability in computational demand, likely due to differences in sequence length and convergence. This burden arises from LSTM's sequential architecture, which captures temporal dependencies through recurrent connections, and the intensive computation required during multiple forward and backward passes across training iterations and hyperparameter optimization [43]. However, testing times were significantly lower (0.2 s), as only a single feedforward pass was required, imposing minimal computational load compared to the iterative training process. Similar discrepancies between training and testing times were reported by Huang et al. [56], who applied an LSTM to predict daily outputs from a carbonate reservoir in the Middle East, with training time of 452.3 s and testing time of 26.95 s.

**Table 4:** Computational cost of model training and inference. Summary statistics for wall-clock time and memory usage for ADM1-R3, random forest (RF), and long short-term memory (LSTM) models during training and testing. For memory, “Peak” denotes the maximum observed usage.

Metric (unit)	Dataset	Statistic	ADM1-R3	RF	LSTM
Time (s)	Training	Mean	3.9	45.9	552.8
		Standard deviation	0.1	1.9	123.1
	Testing	Mean	3.5	0.1	0.2
		Standard deviation	0.3	0.004	0.03
Memory (MB)	Training	Peak	–	7.3	1588.2
		Mean	1.1	3.7	552.2
		Standard deviation	1.0	2.6	223.0
	Testing	Peak	–	4.8	8.4
		Mean	0.9	3.3	4.9
		Standard deviation	1.2	1.7	1.0

Random forest exhibited a significantly shorter training time (45.9 s) than LSTM due to its parallel decision tree construction, which contrasts with the sequential nature of LSTM. Its testing time was the lowest among the three models (0.1 s), as predictions required only traversing pre-built trees [57]. In contrast, ADM1-R3 demonstrated similar training (3.9 s) and testing (3.5 s) times, suggesting a relatively stable computational load across phases. This stability was likely due to the predefined differential equations and the estimated parameters being close to the default values from ADM1-R3, unlike the ML models, which required iterative optimization and model building.

In terms of memory consumption, LSTM demonstrated the highest average RAM usage during training (552.2 MB), significantly exceeding that of RF (3.7 MB) and ADM1-R3 (1.1 MB) (Table 4). This trend aligns with time consumption patterns, reflecting the trade-off between enhanced predictive accuracy and increased computational demands in deep learning models [58]. During testing, LSTM's memory usage declined sharply to 4.9 MB, reflecting reduced resource demands compared to training. Similar to time consumption, this reduction is attributed to the absence of backpropagation and gradient updates during inference [43]. Furthermore, the computational efficiency of LSTMs is highly dependent on the model structure, including the number of layers, hidden units, and sequence length. While increasing these parameters or incorporating optimization algorithms (i.e., GA) can improve the model's ability to capture complex temporal dependencies, it can also significantly increase computational costs [57].

Similarly, RF exhibited relatively stable memory usage between training (3.7 MB) and testing (3.3 MB), reflecting its inherent structure. Training involves constructing multiple decision trees independently, while inference consists of passing data through these pre-built trees, resulting in comparable memory demands across phases. However, RF's computational efficiency is also influenced by hyperparameters, particularly the number of trees in the ensemble [59,60]. While increasing the number of trees may improve predictive accuracy, it also increases memory usage and computational time due to the need to store and process additional decision trees.

Finally, ADM1-R3 maintained consistently low memory consumption during both the calibration (2023) and validation (2024) phases, suggesting that its computational complexity arises primarily from its dependence on extensive biochemical input data and the numerical solution of ordinary differential equations, rather than from intensive processing demands. In summary, LSTM incurred the highest computational cost but achieved the best *NSE*, even with simpler input features. RF demonstrated significantly lower time and memory requirements, making it suitable for applications requiring rapid predictions with moderate accuracy. Meanwhile, ADM1-R3 exhibited the greatest computational efficiency but relied heavily on detailed biochemical characterization, which may not always be available.

Evaluating model performance based on computational resource consumption and time requirements provides a more comprehensive assessment of their capabilities, which can impact the decision-making process in model selection [43]. However, a direct comparison between ADM1-R3 and the ML models is constrained by differences in software environments (MATLAB vs. Python), limiting the extent to which their computational performance can be directly compared. Consequently, the findings primarily indicate broad trends rather than absolute performance disparities. Nevertheless, this analysis enables a more direct comparison between RF and LSTM, offering insights into their relative computational demands. It should be noted that, for this research, the execution time and memory usage were thoroughly measured and compared across all models. However, the absolute differences in training and testing times and memory requirements remain low, and therefore, in practice, model selection would not rely solely on these metrics. Ultimately, the selection between data-driven approaches and mechanistic models will depend on the specific research objectives. If a detailed understanding of the AD process and mechanistic insights are required, ADM1 remains the most appropriate choice despite its extensive data requirements. Conversely, if the primary aim is to develop a predictive tool with minimal experimental effort, ML algorithms may offer a more practical and computationally efficient alternative, despite their relatively higher resource consumption and ‘black-box’ nature.

## 4 Conclusions

This study examined the application of a simplified ADM1 and two ML algorithms, RF and LSTM, to simulate biogas and methane production at a full-scale biogas plant co-digesting different agricultural feedstocks. Based on available measurements, different input combinations were tested within the ML algorithms to identify the minimum set of inputs required to achieve predictive accuracy comparable to ADM1. Results indicated that the three models achieved comparable *NSE* values in simulating biogas and methane production. LSTM achieved the highest *NSE* using only feedstock quantities (t d^−1^) as input features, whereas RF further benefited from including OLR and VS flow from maize silage. Maize silage, in terms of both fresh (t d^−1^) and VS (t VS d^−1^) flow, was identified as the most important feature for the ML models. LSTM demonstrated the relatively highest computational demand, with training times 11 times longer than RF and 141 times longer than ADM1-R3, underscoring a potential trade-off between computational cost, model efficiency, and extensive input requirements. Consequently, model selection should be guided by the study's specific objectives, such as prioritizing predictive accuracy with simplified input variables (ML) or conducting detailed simulations of the AD process based on biochemical pathways (ADM1-R3). These findings contribute to advancing modelling approaches for full-scale agricultural biogas production systems. The methodology developed in this study will enhance the accuracy of biogas yield predictions from full-scale AD digesters.

## CRediT authorship contribution statement

**Sofia Tisocco:** Writing – original draft, Software, Methodology, Investigation, Formal analysis, Data curation, Conceptualization. **Sören Weinrich:** Writing – review & editing, Software, Methodology, Conceptualization. **Henrik Bjarne Møller:** Writing – review & editing, Supervision. **Alastair James Ward:** Writing – review & editing. **Liam Kilmartin:** Writing – review & editing, Software. **Xinmin Zhan:** Writing – review & editing, Supervision, Funding acquisition, Conceptualization. **Paul Crosson:** Writing – review & editing, Supervision.

## Declaration of competing interest

The authors declare that they have no known competing financial interests or personal relationships that could have appeared to influence the work reported in this paper.
