# Supplementary material for: Machine learning vs. ADM1: Reliable biogas prediction with minimal data requirements in full-scale plants
Source: Environ Sci Ecotechnol. 2026 Jan 24;29:100662. doi: 10.1016/j.ese.2026.100662 (PMC12874345; doi:10.1016/j.ese.2026.100662)
Supplement: Multimedia component 1 [file mmc1.docx]

# Machine learning vs. ADM1: Reliable biogas prediction with minimal data requirements in full-scale plants

# Sofia Tisocco ^a,b^, Sören Weinrich ^c,d^, Henrik Bjarne Møller ^e^, Alastair James Ward ^e^, Liam Kilmartin ^f^, Xinmin Zhan ^a,g,h^, Paul Crosson ^b^

^a^ Civil Engineering, School of Engineering, University of Galway, Galway, H91 TK33, Ireland

^b^ Teagasc Animal and Bioscience Research Department, Animal and Grassland Research and Innovation Centre, Dunsany, C15 PW93, Ireland

^c^ Faculty of Energy · Building Services · Environmental Engineering, Münster University of Applied Sciences, Stegerwaldstraße 39, 48565 Steinfurt, Germany

^d^ Biochemical Conversion Department, Deutsches Biomasseforschungszentrum gemeinnützige GmbH, Torgauer Straße 116, Leipzig 04347, Germany

^e^ Department of Biological and Chemical Engineering, Aarhus University, Blichers Allé 20, Tjele 8830, Denmark

^f^ Electrical and Electronic Engineering, School of Engineering, University of Galway, Galway, H91 TK33, Ireland

^g^ Ryan Institute, University of Galway, Galway, H91 TK33, Ireland

^h^ MaREI Research Centre for Energy, Climate and Marine, Ryan Institute, University of Galway, Galway, H91 TK33, Ireland

* Corresponding author: xinmin.zhan@universityofgalway.ie

Summary Information

Number of pages: 8

Figures: S1–S4

Tables: S1–S5

**
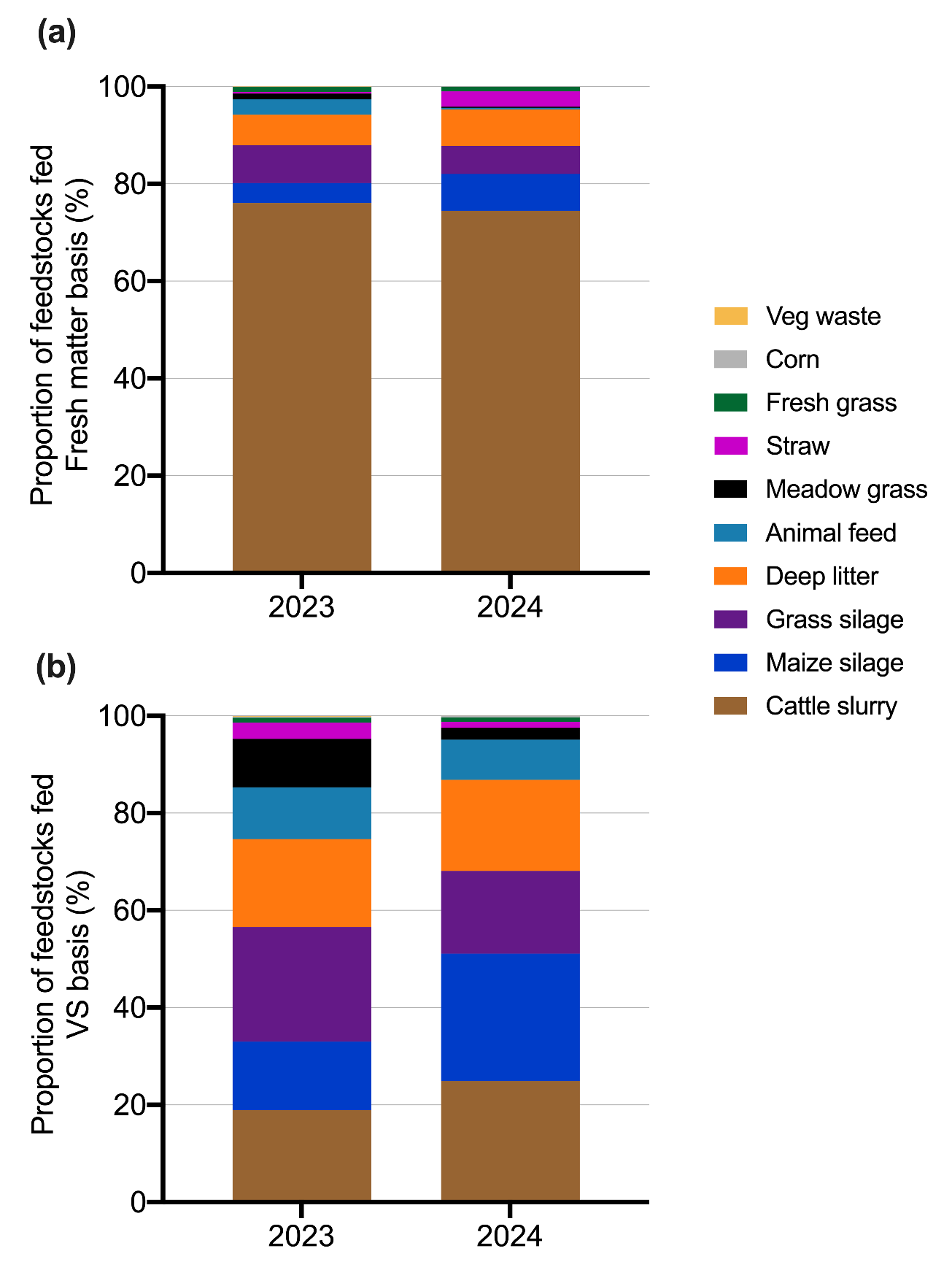
**

**Fig. S1.** Average proportion of feedstocks fed to the biogas plant in (a) fresh matter and (b) volatile solids (VS) basis for 2023 and 2024.


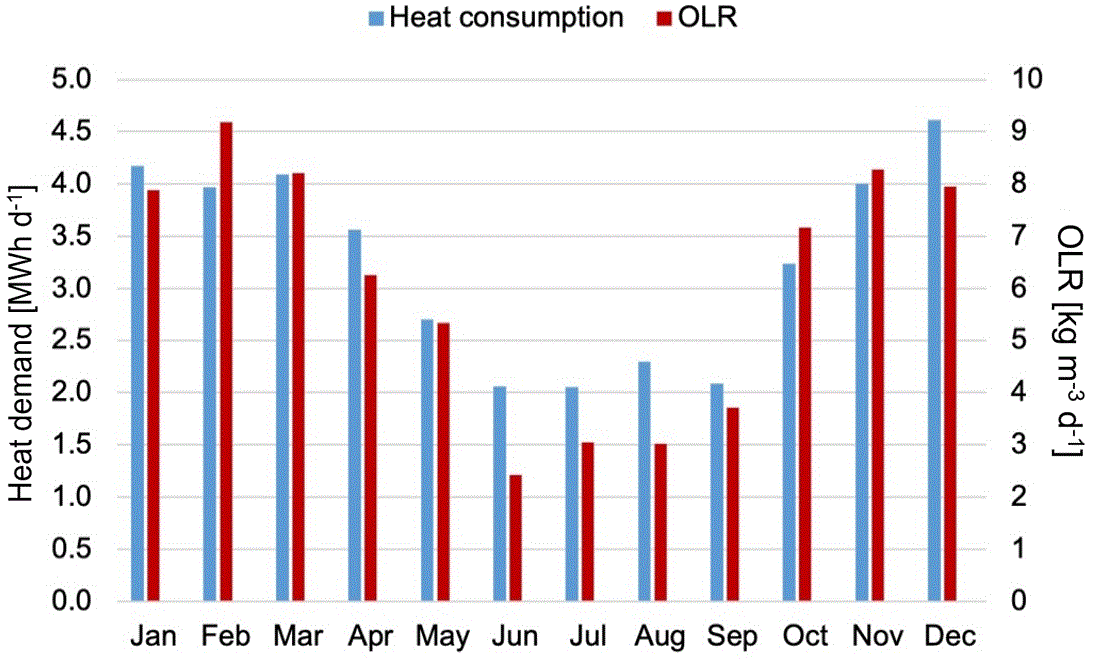


**Fig. S2.** Annual variation in heat consumption at AU Foulum and the corresponding organic loading rate (OLR) adjustments in the biogas plant, calculated based on a primary reactor volume of 1200 m^3^

| **Table S1**  Characteristics of feedstocks used in this study for 2024 ^a^ | | | | |
| --- | --- | --- | --- | --- |
|  | Unit | Maize silage | Grass silage | Deep litter |
| TS | % FM | 28.5 | 18.8 | 28.3 |
| VS | % TS | 96.7 | 89.8 | 80.8 |
| XA | g kg TS^-1^ | 32.6 | 102.2 | 192.0 |
| Total N | g L^-1^ | 3.3 | 5.4 | 6.4 |
| NH_4_-N | g L^-1^ | 0.3 | 2.2 | 1.5 |
| ADF | g kg TS^-1^ | 231.8 | 417.0 | 471.0 |
| NDF | g kg TS^-1^ | 423.1 | 662.7 | 641.4 |
| ADL | g kg TS^-1^ | 26.5 | 74.1 | 123.2 |

^a^ Total solids (TS), crude ash (XA), nitrogen (N), ammonium nitrogen (NH_4_-N), acid detergent fibre (ADF), neutral detergent fibre (NDF), acid detergent lignin (ADL).

| **Table S2**  Procedure for calculating degradable carbohydrates from available measurements ^a^ | | |
| --- | --- | --- |
| Available measurement | Calculation of degradable carbohydrates (DXC) | Reference |
| Acid detergent lignin (ADL) | $DXC=XC-ADL$ | Tisocco et al. (2023) |
| Degradability quotient (DQ) | $DXC=XC*DQ$ | Weinrich and Nelles (2021b) |
| Crude fiber (XF) | $iXC=35+0.47*XF+0.00104*\mathrm{XF}^{2}$ (Maize)  i$XC=35-0.26*XF+0.00300*\mathrm{XF}^{2}$ (Grass)  $DXC=XC-iXC$ | Weißbach (2008) |

^a^ Indigestible crude carbohydrates (iXC)

| **Table S3**  Hyperparameters optimized for each machine learning algorithm | | |
| --- | --- | --- |
| Algorithm | Hyperparameter | Values tested |
| Random forest | Number of estimators | 100; 300; 500; 700 |
|  | Maximum depth of trees | 10; 20; 30; 40; None |
|  | Minimum samples to split | 2; 5; 10; 15 |
|  | Minimum samples per leaf | 1; 2; 4; 6; 10 |
|  | Maximum features for splits | Square root (sqrt); Logarithm base 2 (log2); None |
| Long short-term memory network | Number of units | 32; 64; 128 |
|  | Dropout rate | 0.1; 0.2; 0.3 |
|  | Optimization algorithm | Adaptive moment estimation (Adam); Root mean square propagation (RMSprop) |
|  | Batch size | 16; 32; 64 |
|  | Number of epochs | 20; 50 |

**
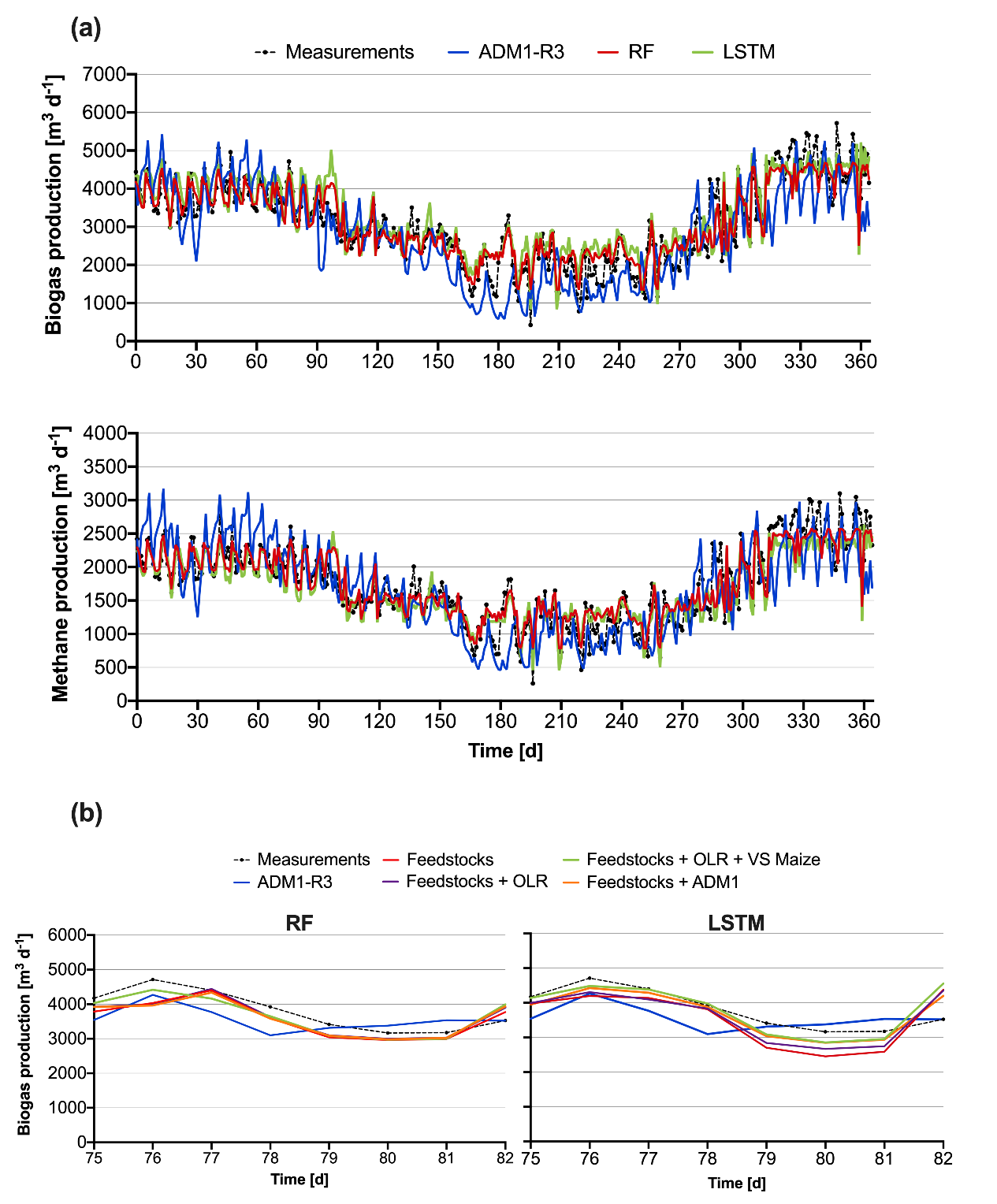
**

**Fig. S3**. (a) Training results (2023) of biogas and methane production from ADM1-R3, RF and LSTM (inputs: feedstocks, OLR and VS from maize silage); (b) Impact of different input combinations on RF and LSTM simulations.

| **Table S4**  Daily and weekly Nash-Sutcliffe efficiency (NSE) for each model output on the training data (2023) | | | |
| --- | --- | --- | --- |
| Model | NSE ^a^ | Biogas production | Methane production |
| ADM1-R3 | Daily | 0.48 | 0.46 |
|  | Weekly | 0.68 | 0.70 |
| Random forest |  |  |  |
| Feedstocks | Daily | 0.83 | 0.85 |
|  | Weekly | 0.92 | 0.93 |
| Feedstocks + OLR | Daily | 0.84 | 0.83 |
|  | Weekly | 0.93 | 0.93 |
| Feedstocks + OLR + VS Maize | Daily | 0.85 | 0.84 |
|  | Weekly | 0.94 | 0.94 |
| Feedstocks + ADM1 inputs | Daily | 0.93 | 0.92 |
|  | Weekly | 0.98 | 0.97 |
| Long short-term memory |  |  |  |
| Feedstocks | Daily | 0.73 | 0.96 |
|  | Weekly | 0.89 | 0.86 |
| Feedstocks + OLR | Daily | 0.73 | 0.96 |
|  | Weekly | 0.89 | 0.88 |
| Feedstocks + OLR + VS Maize | Daily | 0.74 | 0.96 |
|  | Weekly | 0.89 | 0.89 |
| Feedstocks + ADM1 inputs | Daily | 0.76 | 0.96 |
|  | Weekly | 0.90 | 0.90 |

# ^a^ Daily NSE denote daily comparison between measurements and simulation results whereas weekly NSE compare the weekly production sum between measurements and simulation results.

| **Table S5**  Root mean squared error (RMSE) for each model output on the training (2023) and testing (2024) data ^a^ | | | |
| --- | --- | --- | --- |
| Model | RMSE | Biogas production | Methane production |
| ADM1-R3 | Training | 777.9 | 422.8 |
|  | Testing | 905.9 | 499.5 |
| Random forest |  |  |  |
| Feedstocks | Training | 456.3 | 248.4 |
|  | Testing | 897.5 | 509.9 |
| Feedstocks + OLR | Training | 453.1 | 295.6 |
|  | Testing | 866.9 | 493.8 |
| Feedstocks + OLR + VS Maize | Training | 445.1 | 239.1 |
|  | Testing | 857.6 | 479.6 |
| Feedstocks + ADM1 inputs | Training | 423.0 | 231.5 |
|  | Testing | 882.6 | 499.9 |
| Long short-term memory |  |  |  |
| Feedstocks | Training | 558.0 | 313.2 |
|  | Testing | 830.2 | 481.9 |
| Feedstocks + OLR | Training | 557.2 | 295.6 |
|  | Testing | 815.2 | 467.8 |
| Feedstocks + OLR + VS Maize | Training | 554.9 | 295.1 |
|  | Testing | 831.9 | 470.4 |
| Feedstocks + ADM1 inputs | Training | 528.9 | 288.8 |
|  | Testing | 857.6 | 458.0 |

# ^a^ $\mathbf{RMSE=}\sqrt{\frac{\sum_{\mathbf{i}\mathbf{=1}}^{\mathbf{n}} {\mathbf{(}\mathbf{x}_{\mathbf{i}}\mathbf{-}\mathbf{y}_{\mathbf{i}}\mathbf{)}}^{\mathbf{2}}}{\mathbf{n}}}$ , where, *xi* represents the measured values, *yi* denotes the simulation results and *n* is the number of data points (n=365 and n=366 for training and testing data sets, respectively)

#
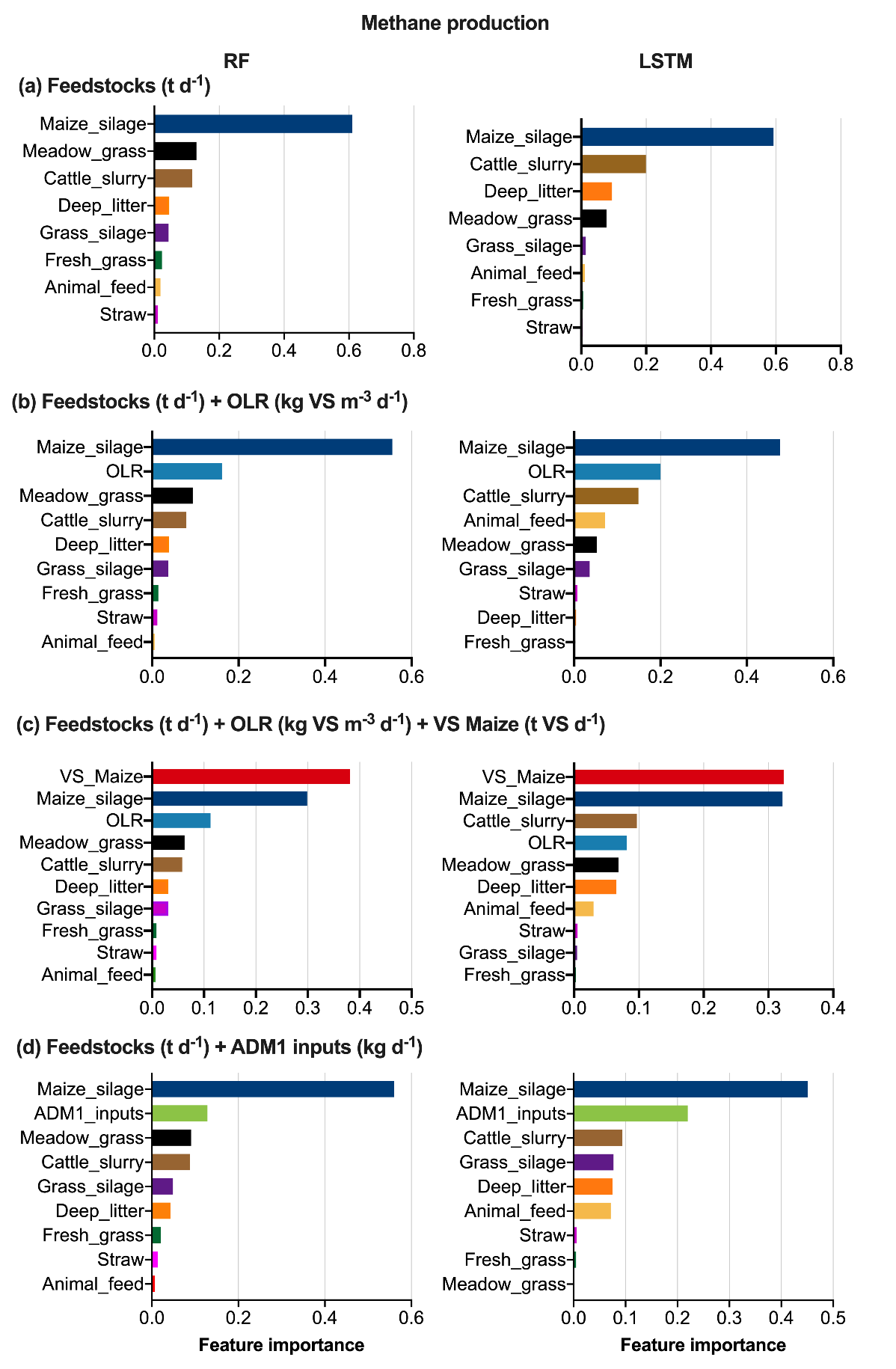


**Fig. S4**. Feature importance of methane simulations (2024) using RF and LSTM using normalized mean |SHAP| values across different input combinations.
